# Investigation of the effects of systemic meperidine administration on fascia healing in an experimental rat model^1^

**DOI:** 10.1590/ACB351107

**Published:** 2020-12-18

**Authors:** Emine Sensoy, Alper Celal Akcan, Mahmut Korkmaz, Ferhan Elmalı, Ugur Topal, Hulya Akgun, Sabahattin Muhtaroglu

**Affiliations:** I MD, Yuksek Ihtisas State Hospital, Department of General Surgery, Kirikkale, Turkey. Conception and design, manuscript preparation and writing.; II Professor, Department of General Surgery, Erciyes University Faculty of Medicine, Melikgazi, Kayseri, Turkey. Conception and design, analysis and interpretation of data, technical procedures, manuscript preparation and writing, critical revision, final approval.; III PhD, Mustafa Cikrikcioglu Vocational School, Department of Tekstille, Erciyes University, Melikgazi, Kayseri, Turkey. Conception and design; acquisition, analysis and interpretation of data; technical procedures, statistics analysis.; IV Associate Professor, Department of Biostatistics, Erciyes University Faculty of Medicine, Melikgazi, Kayseri, Turkey. Conception and design; acquisition, analysis and interpretation of data.; V MD, Department of Surgical Oncology, Erciyes University Faculty of Medicine, Melikgazi, Kayseri, Turkey. Manuscript preparation and writing, final approval.; VI Professor, Department of Pathology, Erciyes University Faculty of Medicine, Melikgazi, Kayseri, Turkey. Conception and design; acquisition, analysis and interpretation of data; histopathological examinations.; VII Professor, Department of Medical Biochemistry, Erciyes University Faculty of Medicine, Melikgazi, Kayseri, Turkey. Conception and design, acquisition of data, technical procedures.

**Keywords:** Wound Healing, Analgesia, Meperidine, Rats

## Abstract

**Purpose:**

To evaluate the effects of meperidine on fascial healing.

**Methods:**

Seventy adult male Sprague-Dawley rats divided into 7 groups with 10 rats in each group. One of these groups was determined as the sham group, 3 of the remaining 6 groups as meperidine groups, and 3 as control groups. These were grouped as 1st, 2nd, and 6th weeks. In the anterior abdominal wall of the rat, the skin was detached and a wound model including the peritoneum was created with a median incision. Mice in the meperidine group were injected with meperidine intraperitoneally (IP) 3 × 20 mg/kg meperidine on postoperative days 0, 1 and 2, and 2 × 20 mg/kg meperidine on postoperative days 3, 4, 5, and 6 after surgical intervention. Similar to the control group, an equal volume of saline was administered, corresponding to the doses. After sacrifice, the midline fascia was used for facial tensile strength measurement, and the other for histopathological analysis.

**Results:**

When compared, the meperidine and control groups inflammatory cell density was higher in the 1st week (p < 0.05) in the meperidine group, fibroplasia density was found to be higher at the 2nd week in the meperidine group than the control group (p < 0.05) When the tensile strength in the meperidine and control groups were compared, there was no significant difference (p > 0.05) at each of the three weeks.

**Conclusion:**

The application of postoperative systemic meperidine affects positively wound healing in the inflammatory stage and fibroplasia without changing the resistance to traction.

## Introduction

Wound healing consists of inflammation, collagen deposition (proliferation), and collagen stages. Stages cannot be separated from each other by sharp boundaries, they develop within each other in an organized way^1^.

Postoperative pain begins with surgical trauma and affects negatively the morbidity and mortality of the patient. In postsurgical pain control, opioids are the most preferred agents, by reducing pain scores and stress response. Especially meperidine, an opioid analgesic, is widely used in Turkey.

Many opioids and their effects on wound healing have been studied in the literature^2^–^5^. In the study conducted by Poonawala *et al*.^2^, it was emphasized that fentanyl, hydromorphone and morphine accelerate healing in ischemic wounds in the experimental rat model.

Many more studies such as these have examined the effects of different opioids on wound healing, depending on duration and dose. There are data with positive and negative findings, but most wound healing studies are skin-focused^2^–^8^. However, for an optimal wound healing, especially in the abdominal wall, the fascial layer, which is the main load bearing part, must be complete. This is of great importance in abdominal surgery.

In the literature, there is no study addressing the effect of meperidine used for postsurgical analgesic on fascial healing. In this study, the effects of opioid derivative meperidine, which is frequently preferred in Turkey for analgesic purposes, were directly and indirectly evaluated for wound healing.

## Methods

After the permission of the Ethics Committee of the Erciyes University Faculty of Medicine dated 16.11.2011 and numbered 11/128, 70 adult male Sprague-Dawley weighing between 250-300 g were studied at the Erciyes University Faculty of Medicine Hakan Çetinsaya Experimental and Clinical Research Center (DEKAM).

The rats were kept in standard plastic cages. Rats were fed with standard rat chow (Aytekinler, Turkey), and tap water was used as drinking water. The environment of the rats was heated to an average of 20 °C and ventilated with air conditioner. Ambient humidity was kept between 40 – 50%. The light system was set up to be 12-hour day, from 7:00 in the morning to 19:00 in the evening, and 12-hour night.

### Study protocol

According to the Rosner power analysis method, the sample size was determined as *n* = 10 in each group due to independent sample and independent parameters in 95% confidence interval. Rats were randomly divided into 7 groups, with 10 rats per group. One of them was separated as the sham group, the remaining 6 groups as 3 meperidine and 3 saline groups.

**Figure 1:**
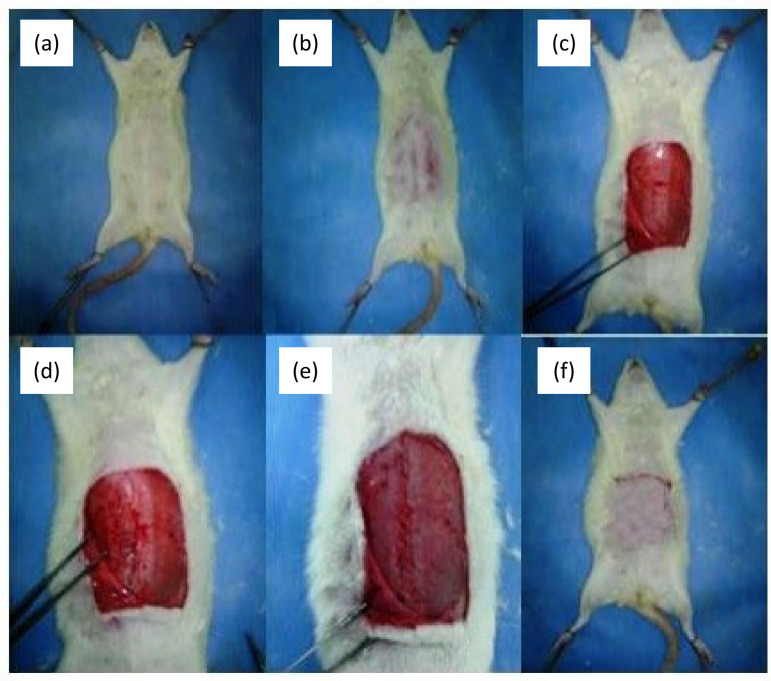
Creation of the wound model. **(a)** Rat is fixated from four limbs. **(b)** The skin is shaved. **(c)** The skin is opened through a paramedian incision, fascia and muscle tissue is reached. **(d)** Fascia is opened with a median incision at linea alba. **(e)** Median incision is closed according to the incision procedure. **(f)** The skin is closed appropriately.

After anesthesia and analgesia were provided to the rats by intraperitoneal 50 mg/kg ketamine hydrochloride (Ketas, EWL Eczacıbaşı Warner Lambert Pharmaceutical Industry and Training LTD, Istanbul) and xylazine hydrochloride (Rhompun, Bayer Pharmaceutical Industry), the skin was detached. A laparotomy was performed with a median incision with an average incision of 5 cm (Fig. 1). Rats in the meperidine group were intraperitoneally injected with 3 × 20 mg/kg meperidine on postoperative days 0, 1 and 2, and 2 × 20 mg/kg meperidine on postoperative days 3, 4, 5 and 6 (Pethidine HCl-Aldolan 2 mL, 100 mg). In the control group, rats were intraperitoneally injected in the volume of saline equivalent to 2 × 20 mg/kg Meperidine on the 1st and 2nd days, and 2 × 20 mg/kg on the 3rd, 4th, 5th and 6th days after surgery. Postoperative antibiotics were not given. Postoperative analgesia was provided for 7 days with meperidine in half of the rats and with saline in the control group. Rats were fed with standard laboratory food and water until the day when they were sacrificed with high dose pentobarbital in the 1st, 2nd, and 6th weeks.

### Histopathological evaluation

After the sacrifice of the rats in both groups at the 1st, 2nd and 6th weeks, a 3 × 3 cm piece was dissected including the previous median abdominal fascia incision, by removing the dermal flap with the old paramedian incision. The received piece was divided into 3 equal pieces of 1 × 3 cm. One of the pieces was fixed in 10% formalin. The fixed specimens were sampled. The sampled pieces were embedded in paraffin blocks after 24 h of tissue follow-up. They were embedded in paraffin blocks appropriately with their longitudinal axes. Preparations were done by taking sections from each sample, each with a thickness of 5–6 microns, including the wound surface. The preparations were evaluated with light microscopy (Olympus BX51) by staining with hematoxylin-eosin to observe inflammatory changes in healing wounds. The histopathological study was carried out by the same pathologist without knowing which tissue sample belongs to which group and random selection from tissue samples (blind evaluation). Tissues were histopathologically evaluated, taking into account inflammatory cell infiltration and cell type, new vessel formation (angiogenesis), fibroblastic activity, and collagen density level. Evaluation score of tissues under light microscope was: none: 0, mild: 1, moderate: 2, severe: 3.

### Biomechanical analysis

Rats that were sacrificed at the 1st, 2nd and 6th weeks after the operation were opened with the old paramedian incision and the subcutaneous tissue was detached. The anterior abdominal wall was extensively excised, including the old median incision. One third of the tissue obtained was removed in strip form. Care was taken to ensure that the tissue strips had the same width and length. Section surface areas were calculated as width × length. Tissue strips were kept fresh in saline for a maximum of 2 h, and freshly studied on the Erciyes University Safiye Çıkrıkçıoğlu Vocational School Textile Department tensiometry device.

**Figure 2:**
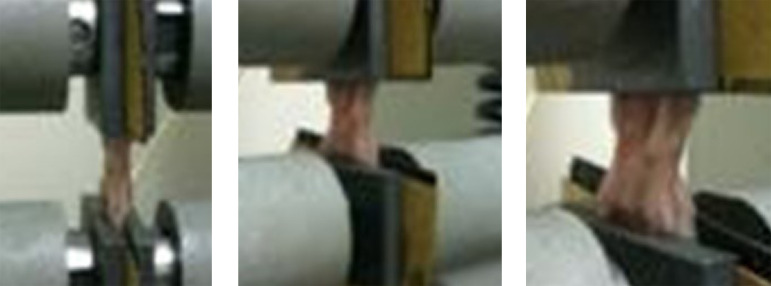
Tissues were placed in the jaws of the device with the incision line in the middle and the tension force was applied at a speed of 10 mm/min until the tissues were broken. The tensile strength was obtained by using the Instron Series IX Automated Material Testing System Version 5.33 device.

The tissue strips were attached from the long axis with the help of a suitable caliper. The distance between the jaws of the Instron brand tensiometry device (TT-CM Model, Instron Eng. Cooperation, Massachusetts, USA) was set to be 20 mm apart and the stretching speed was 10 mm/min. This setting was used in the same way when measuring the stretching force of all tissues. Tissues were placed in the jaws of the device with the incision line in the middle and the tension force was applied at a speed of 10 mm/minuntil the tissues were broken (Fig. 2). Tensile strength was obtained by using the Instron Series IX Automated Material Testing System Version 5.33 device. The tensile strength was recorded in Newton.

### Measurement of hydroxyproline concentrationin tissue

After the rats were sacrificed at the 1st, 2nd, and 6th weeks, one piece of 3 equal-sized strips obtained was removed, containing only the incision in the middle, and prepared in 1 × 1 cm pieces. Tissue samples were washed with saline solution. They were dried with filter paper and placed in the Eppendorf tube. They were stored at 70 °C until the day of the study. Tissues were homogenized in distilled H_2_O, to 100 μL of H_2_O per 10 mg of tissue. One hundred microliters of concentrated HCL (12N) was put into 100 μL of homogenate in a pressure resistant tube with a Teflon cap. It is expected to hydrolyze at 120 °C in 3 h. The hydrolysate obtained was measured using the Biovision Eliza Kit.

### Statistical evaluation

Average, standard deviation, ratio and frequency values were used in the descriptive statistics of the data. The distribution of variables was checked with the Kolmogorov–Smirnov test. In the analyses of angiogenesis, inflammatory cell density, fibroplasia and collagen parameters, which do not show normal distribution, Mann–Whitney U test was used in binary groups, while Kruskal–Wallis tests in more than two groups. Hydroxyproline and tensile strength (Newton) parameters showing normal distribution are independent student t-test in two groups. One-way ANOVA tests were used in more groups. Bonferroni method, one of the post hoc analyzes, was used to determine the source of difference between groups in more than two groups. SPSS 21.0 program was used in the analysis. According to the results of the analysis, the situations where p < 0.05 were considered statistically significant.

## Results

In this study, the meperidine and the saline (control) groups at the 1st, 2nd, and 6th weeks, and both groups at the 1st, 2nd, and 6th weeks were separately evaluated for each parameter.

In the meperidine group, angiogenesis values did not differ significantly (p > 0.05) in subjects of week 1, week 2 and week 6. Other parameters differed significantly (p < 0.05) in subjects of week 1, week 2 and week 6 (Table 1). In the control group, angiogenesis value was significantly higher (p < 0.05) in the 2nd-week group than in the 1st- and 6th-week groups. Other parameters are shown in Table 2.

**Table 1:** Meperidine group by weeks.

Variable	1st week	2nd week	6th week	P
Angiogenesis (x^2^)	2.10 (1–3)	2.20 (1–3)	2.10 (1–3)	0.189
Inflammatory Cell Density (x^2^)	2.00 (1–3)	2.10 (1–3)	1.30 (1–3)***	0.023
Fibroplasia (x^2^)	1.70 (1–3)**	2.10 (1–3)	1.30 (0–2)**	0.001
Collagen (x^2^)	1.50 (1–2)**	2.60 (2–3)	1.10 (0–2)**	0.001
Hydroxyproline (F)	4.10 ± 3.24	2.73 ± 1.37	6.00 ± 2.29**	0.019
Tensile strength (Newton) (F)	8.83 ± 1.38	11.10 ± 2.16	17.38 ± 4.00***	0.000

Kruskal–Wallis (Mann–Whitney U test) / ANOVA (Tukey’s test); Post hoc Bonferroni test, x^2^: Med(Min-Max), F: mean±sd. *p < 0.05 Difference with 1st week /

** p < 0.05 Difference with 2nd week.

**Table 2:** Saline group by weeks.

Variable	1st Week	2nd Week	6th Week	p
Angiogenesis (x^2^)	2.00 (0–3)	3.00 (3–3)*	2.70 (2–3)*	0.003
Inflammatory Cell Density (x^2^)	1.00 (0–2)	1.90 (1–3)*	1.40 (1–2)**	0.001
Fibroplasia (x^2^)	1.40 (1–2)	2.00 (1–3)	1.90 (1–3)	0.092
Collagen (x^2^)	1.10 (0–2)	1.60 (1–3)	1.30 (1–2)***	0.070
Hydroxyproline (F)	5.66 ± 0.98	5.84 ± 2.03	3.71 ± 1.90	0.016
Tensile strength (Newton) (F)	8.24 ± 0.79	9.43 ± 1.72	15.57 ± 4.34***	0.000

Kruskal–Wallis (Mann–Whitney U test) / ANOVA (Tukey’s test); Post hoc Bonferroni test, x^2^: Med(Min-Max), F: mean±sd.

* p < 0.05 Difference with 1st week /

** p < 0.05 Difference with 2nd week.

When the meperidine and control groups were compared, angiogenesis 6th week value was higher in the control group (p < 0.05), inflammatory cell density was higher in the 1th week (p < 0.05) in the meperidine group, fibroplasia density was found to be higher at the 2nd week in the meperidine group than the control group (p < 0.05), and the meperidine group was lower at the 6th week than the control group (p < 0.05). Collagen density did not differ significantly at the 1st, 2nd, and 6th weeks (p > 0.05). The hydroxyproline value was lower at the 2nd week in the meperidine group (p < 0.05) and the hydroxyproline value was lower at the 6th week in the control group (p < 0.05). When the tensile strength in the meperidine and control groups were compared, there was no significant difference (p > 0.05) at each of the three weeks. Comparison of the parameters between the groups is shown in Table 3.

**Table 3:** Comparison of the groups.

Variable	Weeks	Meperidine	Saline	p
Angiogenesis (u)	1st Week	2.10 (1–3)	2.00 (0–3)	0.362
	2nd Week	2.20 (1–3)	3.00 (3–3)	0.481
	6th Week	2.10 (1–3)	2.70 (2–3)	0.021
Inflammatory Cell Density (u)	1st Week	2.00 (1–3)	1.00 (0–2)	0.023
	2nd Week	2.10 (1–3)	1.90 (1–3)	0.781
	6th Week	1.30 (1–3)	1.40 (1–2)	0.302
Fibroplasia (u)	1st Week	1.70 (1–3)	1.40 (1–2)	0.436
	2nd Week	2.10 (1–3)	2.00 (1–3)	0.007
	6th Week	1.30 (0–2)	1.90 (1–3)	0.048
Collagen (u)	1st Week	1.50 (1–2)	1.10 (0–2)	0.123
	2nd Week	2.60 (2–3)	1.60 (1–3)	0.123
	6th Week	1.10 (0–2)	1.30 (1–2)	0.280
Hydroxyproline (t)	1st Week	4.10 ± 3.24	5.66 ± 0.98	0.162
	2nd Week	2.73 ± 1.37	5.84 ± 2.03	0.001
	6th Week	6.00 ± 2.29	3.71 ± 1.90	0.026
Tensile strength (Newton) (u)	1st Week	8.83 ± 1.38	8.24 ± 0.79	0.260
	2nd Week	11.10 ± 2.16	9.43 ± 1.72	0.072
	6th Week	17.38 ± 3.99	15.57 ± 4.34	0.345

* (u): Mann-Whitney U test,** (t) independent samples t-test, u: Med(Min-Max), t: mean±sd.

## Discussion

Wound healing is a set of events triggered in the organism following damage to ensure body integrity. Wound healing is an issue that has been important since the existence of humanity, supported by continuous studies, but has not yet been fully clarified.

Pain is a component that has multifactorial effects on wound healing. There are many studies in the literature evaluating the effect of opioids topically and systemically on wound healing^7^–^9^. Although it has been generally focused on the skin, opioid and wound healing has been evaluated in other tissues such as cornea and ligaments.

The inflammatory phase of wound healing ends within the first 24 – 48 h. The proliferative phase that approximately begins on the 2nd or 3rd days, lasts 2 – 3 weeks. In this study, inflammatory cells at the wound site in the meperidine group were found intensely in the first 2 weeks, but decreased significantly in the 6th week. In the first week, the inflammatory cell density increased statistically significantly in the meperidine group compared to the control group. We think that meperidine may have done this with the cytokine-like effect, by collecting immune cells to the wound site. Maybe more intense inflammatory process be positive, but wound healing should be considered a process as a whole, not the inflammatory stage alone.

In a study in the literature, it was emphasized that fentanyl, hydromorphone and morphine accelerate healing in ischemic wounds in an experimental rat model. It has been shown that the cell (nucleus) density seen in granulation tissue increases 1.5 – 2.5 times, and angiogenesis increases by 45 – 87%^2^. Similarly, in the present study, cell density increased statistically significantly in the first week of meperidine administration. However, the difference in cell density in the control group was rapidly closed in 2 weeks, but it was higher in the meperidine group. In the sixth week, as expected in wound healing, cell density decreased and it was high in the control group compared to the meperidine group, without statistical significance. This made us think that this increased cell density was faster during the use of meperidine, but its effect continued in the following week. In contrast, in the literature, it has been reported that morphine can disrupt monocyte chemotaxis, hence macrophage formation and cytolytic activities of natural killer cells, and proliferation of lymphocytes due to mitogenic effects^10^–^12^.

Fibroplasia develops with the proliferation of fibroblasts in the proliferative stage of wound healing^13^. In vitro study, it was concluded that nalbuphine, a semisynthetic opioid, damages stromal fibroblasts and corneal epithelium, thereby adversely affecting wound healing^14^. In another study, it was observed that the number of myofibroblasts and macrophages decreased in the tissue samples taken in the proliferative phase in burned morphine-addicted rats^6^. In the present study, unlike other studies, fibroplasia increased statistically significantly in the proliferative phase, especially in the 2nd week. We can attribute this to meperidine increasing inflammatory cell migration and density starting from the first week. More cells produced more cytokines, causing a large number of fibroblasts to mobilize from adjacent connective tissue, which may result in more intense fibroplasia.

It has been emphasized that dalargin, an opioid peptide, increases fibroblast proliferation up to three times, increases capillary proliferation, accelerates the maturation of granulation tissue and scar tissue, increases epithelialization and shortens the duration of all these healing periods^7^. Similarly, fibroplasia increased in the present study.

Increasing density of fibroplasia in this study suggests that collagen, hydroxyproline and tensile strength should be high in the same stages. As expected, the value of collagen was significantly high at the 2nd week. However, while hydroxyproline value was expected to increase in parallel with these, it did not increase. The tensile strength was higher than the control group, but was not statistically significant.

Collagen is the most important protein of connective tissue. Collagen synthesis accelerates in normal wound healing in the 2nd week and the synthesis decreases after the 4th week. In parallel with the present study, the density of collagen was significantly higher in the first 2 weeks in the group when applied meperidine and in control group. At the 6th week, collagen density decreased both groups.

Angiogenesis begins on the 3rd and 4th postoperative days and continues throughout the proliferative phase. In many studies, opioids have been shown to increase angiogenesis^14^–^16^. In the meperidine group, the angiogenesis values did not differ significantly (p > 0.05) in the 1st, 2nd and 6th week subjects. However, in the control group, the angiogenesis value in the 1st-week group was significantly lower than the 2nd- and 6th-week groups (p < 0.05). When compared, the angiogenesis value in the meperidine and control groups in the 1st and 2nd week did not differ significantly (p > 0.05), while the angiogenesis value was significantly higher in the control group than in the meperidine group (p < 0.05) at the 6th week. Angiogenesis does not appear to be increased in the present study. In this study, the absence of significant changes between the control and study groups in the evaluation of angiogenesis may suggest that meperidine does not contribute much to the formation of new vessels or does not show its angiogenic effects. In addition, angiogenesis increases in the 6th week and the infiltration of cells is more, it can be considered in favor of prolongation of the inflammatory process. However, to evaluate this, there is a need for experimental studies with other tissues where bleeding is better than fascia.

Hydroxyproline is the most important component of collagen. The amount and storage of collagen after trauma increases in the first 2 weeks in the proliferative phase and decreases after the 4th week. In this study, when meperidine and control groups were compared, there was no significant difference (p > 0.05) in the 1st week, while the meperidine group was significantly lower than the control group (p < 0.05) at the 2nd week. On the 6th week, meperidine group was significantly higher than the control group (p < 0.05). This fact may be because we calculate hydroxyproline measurement calorimetrically, but subjectively evaluate collagen from histological sections. Because in our evaluation of collagen density, the collagen density is higher in the meperidine group. In addition, a technical deficiency may be the reason for this development.

The bursting force is the force that causes the wound edges to open. In the first week, the wound tension force is 3% of the final strength of the wound, 10% in the second week, and 30% at the end of 1 month^8^. While 50% strength of normal tissue is achieved in around 3 months, only 80% tension force of normal tissue is obtained when maturation is completed.

In the present study, the tensile strength was significantly higher (p < 0.05) in the study and control groups in the 6th week compared to the 1st and 2nd weeks. There was no significant difference (p > 0.05) between the 1st- and 2nd-week groups. When the control and meperidine groups were compared, the meperidine group was found to have higher scores in the 1st, 2nd, and 6th weeks, but this was not statistically significant. However, the amount of meperidine used in higher doses for a longer period of time may cause different results.

Meperidine strengthened the proliferative phase of wound healing, causing more fibroplasia, and accelerated collagen production. However, a significant increase in the tensile strength, which is expected to show the integrity of the wound, was not achieved in parallel with these. The reason for this may be that the fibroblasts were not able to produce the necessary chemokine and lymphokine stimulus despite the high density of inflammatory cells in the first week. Perhaps the number of neuropeptides that act as a cytokine and increase wound healing may have decreased due to the given meperidine.

## Conclusion

Although meperidine, which use as intraperitoneally and in high doses for analgesic purposes, increases the density of inflammatory and fibroblastic cells that we evaluate as wound healing scores, it did not significantly increase the tensile strength (p > 0.05), which is the end result of wound healing.
